# How do 66 European institutional review boards approve one protocol for an international prospective observational study on traumatic brain injury? Experiences from the CENTER-TBI study

**DOI:** 10.1186/s12910-020-00480-8

**Published:** 2020-05-12

**Authors:** Marjolein Timmers, Jeroen T. J. M. van Dijck, Roel P. J. van Wijk, Valerie Legrand, Ernest van Veen, Andrew I. R. Maas, David K. Menon, Giuseppe Citerio, Nino Stocchetti, Erwin J. O. Kompanje, The CENTER-TBI investigators and participants, Cecilia Åkerlund, Krisztina Amrein, Nada Andelic, Lasse Andreassen, Audny Anke, Anna Antoni, Gérard Audibert, Philippe Azouvi, Maria Luisa Azzolini, Ronald Bartels, Pál Barzó, Romuald Beauvais, Ronny Beer, Bo-Michael Bellander, Antonio Belli, Habib Benali, Maurizio Berardino, Luigi Beretta, Morten Blaabjerg, Peter Bragge, Alexandra Brazinova, Vibeke Brinck, Joanne Brooker, Camilla Brorsson, Andras Buki, Monika Bullinger, Manuel Cabeleira, Alessio Caccioppola, Emiliana Calappi, Maria Rosa Calvi, Peter Cameron, Guillermo Carbayo Lozano, Marco Carbonara, Simona Cavallo, Giorgio Chevallard, Arturo Chieregato, Giuseppe Citerio, Iris Ceyisakar, Mark Coburn, Jonathan Coles, Jamie D. Cooper, Marta Correia, Amra Čović, Nicola Curry, Endre Czeiter, Marek Czosnyka, Claire Dahyot-Fizelier, Paul Dark, Helen Dawes, Véronique De Keyser, Vincent Degos, Francesco Della Corte, Hugo den Boogert, Bart Depreitere, Đula Đilvesi, Abhishek Dixit, Emma Donoghue, Jens Dreier, Guy-Loup Dulière, Ari Ercole, Patrick Esser, Erzsébet Ezer, Martin Fabricius, Valery L. Feigin, Kelly Foks, Shirin Frisvold, Alex Furmanov, Pablo Gagliardo, Damien Galanaud, Dashiell Gantner, Guoyi Gao, Pradeep George, Alexandre Ghuysen, Lelde Giga, Ben Glocker, Jagoš Golubovic, Pedro A. Gomez, Johannes Gratz, Benjamin Gravesteijn, Francesca Grossi, Russell L. Gruen, Deepak Gupta, Juanita A. Haagsma, Iain Haitsma, Raimund Helbok, Eirik Helseth, Lindsay Horton, Jilske Huijben, Peter J. Hutchinson, Bram Jacobs, Stefan Jankowski, Mike Jarrett, Ji-yao Jiang, Faye Johnson, Kelly Jones, Mladen Karan, Angelos G. Kolias, Erwin Kompanje, Daniel Kondziella, Evgenios Koraropoulos, Lars-Owe Koskinen, Noémi Kovács, Ana Kowark, Alfonso Lagares, Linda Lanyon, Steven Laureys, Fiona Lecky, Didier Ledoux, Rolf Lefering, Valerie Legrand, Aurelie Lejeune, Leon Levi, Roger Lightfoot, Hester Lingsma, Andrew I. R. Maas, Ana M. Castaño-León, Marc Maegele, Marek Majdan, Alex Manara, Geoffrey Manley, Costanza Martino, Hugues Maréchal, Julia Mattern, Catherine McMahon, Béla Melegh, David Menon, Tomas Menovsky, Benoit Misset, Davide Mulazzi, Visakh Muraleedharan, Lynnette Murray, Ancuta Negru, David Nelson, Virginia Newcombe, Daan Nieboer, József Nyirádi, Otesile Olubukola, Matej Oresic, Fabrizio Ortolano, Aarno Palotie, Paul M. Parizel, Jean-François Payen, Natascha Perera, Vincent Perlbarg, Paolo Persona, Wilco Peul, Anna Piippo-Karjalainen, Matti Pirinen, Horia Ples, Suzanne Polinder, Inigo Pomposo, Jussi P. Posti, Louis Puybasset, Andreea Radoi, Arminas Ragauskas, Rahul Raj, Malinka Rambadagalla, Jonathan Rhodes, Sylvia Richardson, Sophie Richter, Samuli Ripatti, Saulius Rocka, Cecilie Roe, Olav Roise, Jonathan Rosand, Jeffrey V. Rosenfeld, Christina Rosenlund, Guy Rosenthal, Rolf Rossaint, Sandra Rossi, Daniel Rueckert, Martin Rusnák, Juan Sahuquillo, Oliver Sakowitz, Renan Sanchez-Porras, Janos Sandor, Nadine Schäfer, Silke Schmidt, Herbert Schoechl, Guus Schoonman, Rico Frederik Schou, Elisabeth Schwendenwein, Charlie Sewalt, Toril Skandsen, Peter Smielewski, Abayomi Sorinola, Emmanuel Stamatakis, Simon Stanworth, Robert Stevens, William Stewart, Ewout W. Steyerberg, Nino Stocchetti, Nina Sundström, Anneliese Synnot, Riikka Takala, Viktória Tamás, Tomas Tamosuitis, Mark Steven Taylor, Braden Te Ao, Olli Tenovuo, Alice Theadom, Matt Thomas, Dick Tibboel, Marjolein Timmers, Christos Tolias, Tony Trapani, Cristina Maria Tudora, Peter Vajkoczy, Shirley Vallance, Egils Valeinis, Zoltán Vámos, Gregory Van der Steen, Joukje van der Naalt, Jeroen T. J. M. van Dijck, Thomas A. van Essen, Wim Van Hecke, Caroline van Heugten, Dominique Van Praag, Thijs Vande Vyvere, Roel P. J. van Wijk, Alessia Vargiolu, Emmanuel Vega, Kimberley Velt, Jan Verheyden, Paul M. Vespa, Anne Vik, Rimantas Vilcinis, Victor Volovici, Nicole von Steinbüchel, Daphne Voormolen, Petar Vulekovic, Kevin K. W. Wang, Eveline Wiegers, Guy Williams, Lindsay Wilson, Stefan Winzeck, Stefan Wolf, Zhihui Yang, Peter Ylén, Alexander Younsi, Frederick A. Zeiler, Veronika Zelinkova, Agate Ziverte, Tommaso Zoerle

**Affiliations:** 1 Department of Intensive Care, Erasmus MC - University Medical Centre Rotterdam, P.O. Box 2040, 3000 CA Rotterdam, the Netherlands; 2 Department of Neurosurgery, University Neurosurgical Center Holland, LUMC, HMC & Haga Teaching Hospital, Leiden, The Hague The Netherlands; 3 ICON plc, South County Business Park Leopardstown, Dublin 18, Ireland; 4 Department of Public Health, Erasmus MC – University Medical Centre Rotterdam, Rotterdam, the Netherlands; 5 Department of Neurosurgery, Antwerp University Hospital, Edegem, Belgium; 6 University of Antwerp, Antwerp, Belgium; 7 Department of Anaesthesia, University of Cambridge, Cambridge, UK; 8 School of Medicine and Surgery, University of Milan-Bicocca, Milan, Italy; 9 San Gerardo Hospital, ASST-Monza, Monza, Italy; 10 Department of Physiopathology and Transplantation, Milan University, Milan, Italy; 11 Neuro ICU Fondazione IRCCS Cà Granda Ospedale Maggiore Policlinico Milano, Milan, Italy; 12 Department of Medical Ethics and Philosophy of Medicine, Erasmus MC – University Medical Center Rotterdam, Rotterdam, the Netherlands

**Keywords:** Research ethic committees, European Union, Health-care research, CENTER-TBI, Harmonization

## Abstract

**Background:**

The European Union (EU) aims to optimize patient protection and efficiency of health-care research by harmonizing procedures across Member States. Nonetheless, further improvements are required to increase multicenter research efficiency. We investigated IRB procedures in a large prospective European multicenter study on traumatic brain injury (TBI), aiming to inform and stimulate initiatives to improve efficiency.

**Methods:**

We reviewed relevant documents regarding IRB submission and IRB approval from European neurotrauma centers participating in the Collaborative European NeuroTrauma Effectiveness Research in Traumatic Brain Injury (CENTER-TBI). Documents included detailed information on IRB procedures and the duration from IRB submission until approval(s). They were translated and analyzed to determine the level of harmonization of IRB procedures within Europe.

**Results:**

From 18 countries, 66 centers provided the requested documents. The primary IRB review was conducted centrally (*N* = 11, 61%) or locally (*N* = 7, 39%) and primary IRB approval was obtained after one (*N* = 8, 44%), two (*N* = 6, 33%) or three (*N* = 4, 23%) review rounds with a median duration of respectively 50 and 98 days until primary IRB approval. Additional IRB approval was required in 55% of countries and could increase duration to 535 days. Total duration from submission until required IRB approval was obtained was 114 days (IQR 75–224) and appeared to be shorter after submission to local IRBs compared to central IRBs (50 vs. 138 days, *p* = 0.0074).

**Conclusion:**

We found variation in IRB procedures between and within European countries. There were differences in submission and approval requirements, number of review rounds and total duration. Research collaborations could benefit from the implementation of more uniform legislation and regulation while acknowledging local cultural habits and moral values between countries.

## Background

A Research Ethics Committee or Institutional Review Board (collectively referred to as IRB in the remainder of this manuscript) is appointed to review research protocols to ensure their compliance with ethical standards and national laws. IRBs have an essential role in (clinical) research to protect the dignity, fundamental rights, safety, and well-being of research participants and their formal approval is compulsory before a clinical study can start [1]. Although several international models exist to improve the harmonization of ethical principles, the functioning of IRBs are subject to national legislation and regulation, which refine their structure and function to better serve local needs and cultural preferences [2, 3]. Approval of research protocols submitted to IRBs is subject to these differences, which may complicate the conduct of international research.

Managing variations in IRB procedures is important because of the increasing number of research initiatives which involve multiple European Union (EU) Member States [4–6]. Variation could be improved by harmonization of European law, which is the process of creating uniformity in laws, regulations and practices between countries. Regarding research and IRB procedures, lack of procedural harmonization ‘leads to a complex and uncertain framework for ethical review and for participant information consent, resulting in numerous inefficiencies in observational studies’ [7]. Greater procedural harmonization is generally considered desirable, because it could improve quality and efficiency of healthcare research by decreasing costs, increasing statistical validity, [8–10] optimizing data management, [10] allowing choice of relevant and generalizable outcome variables, [9] promoting uniform product safety regulations [8] and minimizing waste of resources due to inefficiencies [8].

Although most IRBs have websites that describe the local submission process and provide access to submission guidelines and forms, up to date systematic information on IRB procedures and their level of harmonization in European health-care research is scarce. We are aware of only one previous meta-analysis on IRB procedures across European countries from 2005 to 2007 that was also related to research involving acutely mentally incapacitated individuals [6]. The Collaborative European Neurotrauma Effectiveness Research in Traumatic Brain Injury (CENTER-TBI) study is a large observational study conducted in many countries across Europe that provides a unique opportunity to assess European IRB policies and procedures [11].

This study aims to improve the efficiency of future research initiatives by quantifying the differences in IRB procedures through analyzing the procedural details, problems and challenges that researchers encountered in obtaining IRB approval for the general research protocol of the CENTER-TBI study.

## Methods

### Study setting

The Collaborative European NeuroTrauma Effectiveness Research in Traumatic Brain Injury (CENTER-TBI, www.center-tbi.eu) Core study is a prospective observational study on traumatic brain injury (TBI), which was conducted between December 2014 and December 2017 in 63 neurotrauma centers across Europe and Israel [11, 12]. The study included patients with TBI of all severities, and aims to improve characterization of TBI, in order to facilitate the development of precision medicine approaches and to identify best practices by using a comparative effectiveness research (CER) approach [11–14]. In the context of the project high-quality Personal Health related Data (PHD) were collected with repositories for neuro-imaging, DNA, and serum biomarkers. Prior to the study start and collection of clinical data, a uniform CENTER-TBI research protocol including all relevant documents was sent to all responsible IRBs to ensure its legal, ethical and statistical soundness and to obtain IRB approval.

A total of 68 centers from 19 countries initially submitted applications for IRB approval. Because this article focuses on IRB approval in Europe, two centers from Israel were excluded from our analysis. The 66 center that participated in this present study are from Austria (*N* = 2), Belgium (*N* = 5), Denmark (*N* = 2), Finland (*N* = 2), France (*N* = 7), Germany (*N* = 4), Hungary (*N* = 3), Italy (*N* = 8), Latvia (*N* = 3), Lithuania (*N* = 2), the Netherlands (*N* = 7), Norway (*N* = 3), Romania (*N* = 1), Serbia (*N* = 1), Spain (*N* = 4), Sweden (*N* = 2), Switzerland (*N* = 1), and the United Kingdom (UK), (*N* = 9). Sixty-one European centers were initiated and actively enrolled patients in the study.

### Data collection and administration

All IRB submission documents, communication records and approval documents were collated per center by the Contract Research Organization, ICON plc (ICON), directly after final approval of IRBs [15]. ICON is a global company operating in the healthcare industry that was responsible for the clinical monitoring of CENTER-TBI data. The received IRB documents were obtained in 15 different languages (Danish, Dutch, English, Finnish, French, German, Hungarian, Italian, Latvian, Lithuanian, Norwegian, Romanian, Serbian, Spanish, and Swedish) and were partly translated before analysis. The authors contacted the principle investigators to obtain additional information to minimize the amount of unclear or missing data. Identifiable information was deleted to protect the privacy of stakeholders. This resulted in a final set of documents, that was analyzed for this study.

### Analyses

We assessed the IRB review procedures by using the final set of documents and aimed to answer the following research questions in order to evaluate differences in obtaining IRB approval (1) Was the study considered to be observational or interventional? (2) Was the research protocol to be submitted to a central IRB or local IRB for primary IRB review and primary IRB approval? (3) Was additional IRB review required after primary IRB approval had already been obtained? If yes, to what extent? (4) How many review rounds were conducted before primary IRB approval was obtained? What were the reasons? (5) What was the time between protocol submission and obtaining the required IRB approval to start the study? The use of ‘primary’ in this context should be interpreted as first in an order and ‘additional’ as second in an order, without including a statement on importance.

To elaborate on the fifth question, we reconstructed six timeframes regarding the primary IRB review procedure: (1) time between protocol submission and primary IRB approval or first IRB reaction, (2) time between first IRB reaction and first reaction of researcher, (3) time between first reaction of researcher and primary IRB approval or second IRB reaction, (4) time between second IRB reaction and second reaction researcher, (5) time between second reaction researcher and primary IRB approval, and (6) total time between protocol submission and primary IRB approval. The existence of these timeframes naturally depended on the actual procedure. Data on any additional IRB review focused only on the duration of this particular review until the required IRB approval was obtained.

In order to assess regional variation, countries were grouped into six regions based on the United Nation geo-scheme: Baltic States (Latvia, and Lithuania), Eastern Europe (Hungary, Romania, and Serbia), Northern Europe (Denmark, Finland, Norway, and Sweden), Southern Europe (Italy, and Spain), the United Kingdom (UK), and Western Europe (Austria, Belgium, France, Germany, the Netherlands, Switzerland) [16]. Incomplete data was marked ‘Missing’ (M) and all timeframes were reported in days.

To determine significant differences between the time from submission till approval of the research protocol between primary local IRBs and primary central IRBs, we performed a Mann-Whitney U test (continuous). Analyses were performed using R version 3.6.0. Finally, a descriptive analysis of questions, comments and answers from both IRB and researcher during the IRB review procedure was performed to summarize the problems and challenges that researchers encountered in obtaining IRB approval. IRB reactions were categorized and reported by their appearance: (1) Procedure, (2) Blood collection and biomarkers, (3) MRI, (4) Privacy and data security, (5) Other.

## Results

A total of 66 neurotrauma centers from 18 countries were included in this analysis. Most centers were located in Western Europe (*N* = 26, 39%) and least in Eastern Europe (*N* = 5, 8%) and the Baltic States (*N* = 5, 8%). Most participating centers were from the UK (*N* = 9), followed by Italy (*N* = 8), The Netherlands and France (*N* = 7) (Table 1). In all countries the local principal investigators were responsible to submit the general CENTER-TBI research protocol for IRB review and IRB approval.

**Table 1:** Baseline study information

Region Country	Centers (N)	Central or local IRB review	IRB decision on study type
Baltic States	**5**		
Latvia	3	Local^a^	Observational
Lithuania	2	Local	Observational
Eastern Europe	**5**		
Hungary	3	Central	Interventional
Romania	1	Local	Observational
Serbia	1	Local	Observational and Interventional
Northern Europe	**9**		
Denmark	2	Central	Observational
Finland	2	Central	Observational
Norway	3	Central	Observational
Sweden	2	Central	Observational
Southern Europe	**12**		
Italy	8	Central	Observational
Spain	4	Local	Observational
United Kingdom	**9**		
United Kingdom	9	Central^b^	Observational
Western Europe	**26**		
Austria	2	Local	Observational
Belgium	5	Central	Observational
France	7	Central	Interventional
Germany	4	Central	Observational
Netherlands	7	Central	Observational with diagnostic interventions
Switzerland	1	Local	Observational

^a^ Latvia has a local review procedure, but, after approval had been obtained for the first center, other centers did not require additional approval

^b^ In the UK, the research protocol had to be submitted to an external national committee that was not associated to the submitting center. After primary approval by this national committee, all centers (including the submitting center) required additional IRB approval

### Observational or interventional

The majority of countries (*N* = 14, 78%) considered the study to be observational, while others judged it to be observational with diagnostic interventions (The Netherlands), interventional (France, Hungary) and observational and interventional (Serbia) (Table 1).

### Primary central or primary local IRB review

**Fig. 1:**
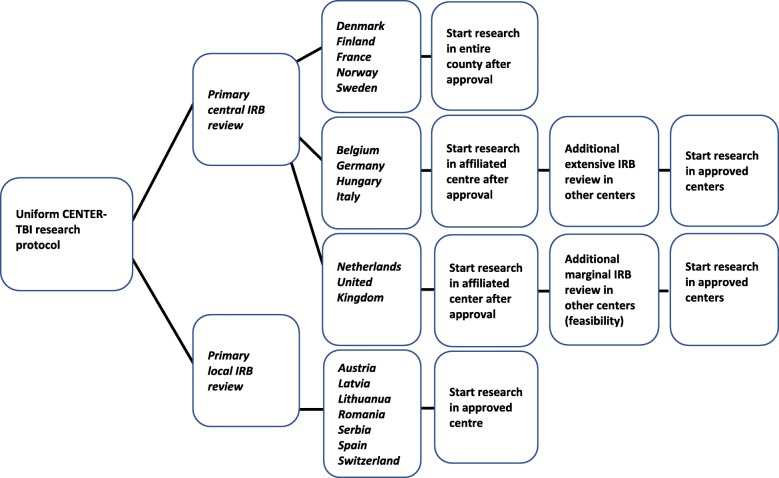
Flowchart of IRB review and approval processes in the CENTER-TBI study. This figure shows an overview of the different IRB review and approval processes in the CENTER-TBI study. IRB; Institutional Review Board

Primary IRB review started directly after protocol submission and was considered ‘central’ when submitted to a central institution or an institution that was part of a national network (*N* = 11, 61%). There were three options: (1) Primary central IRB approval had a national impact and applied to all participating centers within a country, without the need for additional IRB review (*N* = 5; Denmark, Finland, France, Norway, Sweden). (2) Primary central IRB approval only allowed study start in the research centers associated with the approving IRB. Other participating centers in the country required approval after an additional extensive local IRB review. This involved the re-evaluation of the entire protocol and applicable ethics (*N* = 4; Belgium, Germany, Hungary, Italy). (3) Primary central IRB approval only allowed study start in the research centers associated with the approving IRB. Other participating centers required additional approval after marginal local IRB review, mainly assessing local feasibility (*N* = 2; UK, The Netherlands) (Fig. 1).

Primary IRB review was considered ‘local’ when the protocol was submitted to an independent ‘local’ IRB. Obtained primary local IRB approvals only applied to the associated research centers and allowed study start without any additional requirements (*N* = 7; Austria, Switzerland, Spain, Lithuania, Latvia, Romania, Serbia). Primary local IRB review could be performed simultaneously in each independent IRB (Fig. 1).

For every protocol submission, there were two outcome options after IRB review: (1) the required (primary or additional) IRB approval had been obtained and the study could start, or (2) researchers were asked to answer questions or make protocol changes, which was followed by an extra IRB review round. This process varied between IRBs and was repeated until the required IRB approval was eventually obtained. None of the submissions in this study were rejected.

### IRB review rounds

**Fig. 2:**
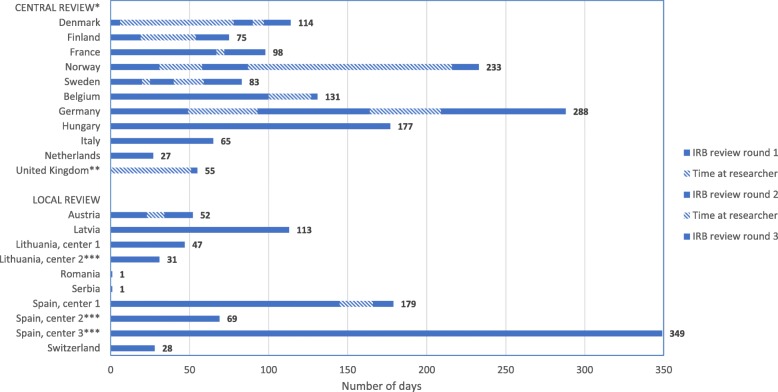
Detailed overview of primary IRB review rounds and duration. This figure provides a detailed overview of the number of primary local and central IRB review rounds and their duration in days. *The number of review rounds was only reported for the initial center of each country. **Information on the first review round was missing. ***Only the total number of days was available

Eight countries (44%), including all countries from Eastern Europe and the Baltic State, obtained primary IRB approval in the first round after submission, while six countries (Austria, Belgium, France, Finland, Spain and UK) required one extra review round and four countries (Denmark, Germany, Norway and Sweden) required two extra review rounds (Fig. 2). Extra review rounds were found in 73% of centers after primary central IRB submission and in 20% after primary local IRB submission.

Several IRBs commented on different aspects of the protocol: selection criteria (*n* = 3, 38%), patient/proxy consent (*n* = 4, 50%), and information forms (*n* = 3, 38%). Also, specific questions were asked on possible non-standard care factors in particular MRI scans (*N* = 4), blood sample collection (*N* = 4). Four questions were asked about privacy and data security, mainly related to the period after study completion. All relevant information can be found in the supplementary files.

### Duration from protocol submission to IRB approval

The median time from protocol submission until the required IRB approval was obtained to start the study was 114 days (IQR 75–224). The fastest required IRB approval was obtained after one day in Serbia and Romania, whereas the longest time was found in a center in the UK (535 days). Obtaining central IRB approval (138 days, IQR: 91–229) took significantly longer (*p* = 0.0074) than obtaining local IRB approval (50 days, IQR: 29–102) (Table 2).

**Table 2:** Duration of protocol submission until required IRB approval before study start

	Duration (days)^a^	Centers (N)	Missing (N)
All centers	114 (75–224)	58	8
Local review	50 (29–102)	10	4
Central review	138 (91–229)^b^	48	4
- Central (1)	98 (94–114)	16	0
- Central (2)	189 (140–270)	17	3
- Central (3)	104 (62–224)	15	1

Local review: Obtained primary local IRB approvals only applied to the associated research centers and allowed study start without any additional requirements

Central (1): Primary central IRB approval with national impact, applying to all center within a country, without the need for additional local IRB review

Central (2): Primary central IRB approval only allowed study start in the research centers associated with the approving IRB. Other participating centers required approval after additional extensive local IRB review

Central (3): Primary central IRB approval only allowed study start in the research centers associated with the approving IRB. Other participating centers required approval after additional marginal local IRB review

^a^Duration was reported in median number of days (IQR)

^b^Group differences between local and central review were significant (P = 0.0074, Mann-Whitney U)

In Norway and Denmark, the majority of time from submission to primary central IRB approval was spent by researchers (67 and 69%, respectively), while in France (95%) and Hungary (71%) most time was consumed by IRBs. Regarding primary local IRB submissions, researchers only accounted for 12% of time in Spain and 21% in Austria (Fig. 2).

Additional IRB review rounds after primary central IRB review were required in 55% of countries. An additional marginal (feasibility) review had a median duration of 104 days (IQR: 62–224), whereas an additional extensive IRB review took 189 days (IQR: 140–270) (Table 3).

**Table 3:** Duration from submission to required IRB approval before study start per country and study center

Country	Central or local IRB review	Duration in days								
		Centre								
		1	2	3	4	5	6	7	8	9
Denmark	Central (1)	114	114							
Finland	Central (1)	75	75							
France	Central (1)	98	98	98	98	98	98	98		
Norway	Central (1)	233	233	233						
Sweden	Central (1)	83	83							
Belgium	Central (2)	131	138	141	257	M				
Germany	Central (2)	288	296	312	M					
Hungary	Central (2)	177	200	204						
Italy	Central (2)	65	70	139	141	155	261	273	288	
Netherlands	Central (3)	27	46	91	209	223	224	M		
United Kingdom^a^	Central (3)	58	61	63	84	104	157	229	282	535
Austria	Local	52	M							
Latvia	Local	113	M	M						
Lithuania	Local	31	47							
Romania	Local	1								
Serbia	Local	1								
Spain	Local	69	179	349	M					
Switzerland	Local	28								

Central (1): Primary central IRB approval with national impact, applying to all center within a country, without the need for additional local IRB review to start study

Central (2): Primary central IRB approval only allowed study start in the research centers associated with the approving IRB. Other participating centers required approval after additional extensive local IRB review to start study

Central (3): Primary central IRB approval only allowed study start in the research centers associated with the approving IRB. Other participating centers required approval after additional marginal local IRB review to start study

Local review: Obtained primary local IRB approvals only applied to the associated research centers and allowed study start without any additional requirements

M = Missing

^a^In the UK, the research protocol had to be submitted to an external national committee not associated to the submitting center. After primary approval by this national committee, all centers required additional IRB approval

Variation between centers within countries was least in Lithuania (31 to 47 days), Germany (288 to 312 days), Belgium (131 to 155 days), and Hungary (177 to 204 days), compared to Spain (69 to 349 days), the Netherlands (27 to 224 days), the UK (58 to 535 days), and Italy (65 to 288 days) (Table 3).

## Discussion

This study shows variation in IRB procedures between and within European countries, indicating a lack of uniform legislation and regulation, or inconsistencies in how such legislation or regulation were implemented. In some countries, a primary central IRB approval was sufficient for study initiation, while others required an additional IRB review at the participating site. Also, the number of review rounds, duration until IRB approval, and the nature of questions and comments from the IRBs varied. Not all IRBs considered the study to be observational, demonstrating a different way of understanding the study. The apparent lack of integration and harmonization in this context suggests that the efficiency of European research collaborations could benefit from improving knowledge on the existing variation in procedures, inefficiencies and differences in value systems between and within countries.

The duration from protocol submission to required IRB approval was highly variable and ranged from one day up to nearly one year. In literature, differences between IRB procedures were also reported and IRB review durations varied from weeks to several months [6, 17]. The difference in total duration between primary central and primary local IRB approval could respectively be overestimated and underestimated by the short primary IRB review times in Serbia and Romania and the missing data of the first review round for the UK. The difference is not necessarily related to the number of review rounds, but might be more explained by the reason and nature (primary central/local review or extensive/marginal additional local review) of the extra review round(s), the accompanying amount of work and the working speed of both IRB and research team. The influence of the latter was substantiated by our data as responding to questions from the IRB seemed to account for an important part of time in several countries (e.g. Denmark and Norway), while the majority of time in other countries (e.g. Belgium, Spain and France) was accounted for by the time taken in primary evaluation by IRBs. The exact reasons for these ‘delays’ could however not be derived from our data and deserves further study. They might be caused by the difficulty of requirements or questions, although, according to the communication records, IRBs mainly requested extra explanation of research procedures. Based on the IRB information requests in this study, special attention should be given to the description of inclusion criteria, informed consent procedures, patient information forms, non-standard care procedures, privacy and data security. A quick response by investigators and agreeing on a maximal turnover time of 1 month to 2 months for IRBs could already minimize substantial delay. This is also in correspondence with literature, where IRB turnover time targets range from 30 to 60 days [17, 18].

The question whether CENTER-TBI was an observational or an interventional study did not appear to be a clear explanation for differences in number and duration of review rounds. Interventional studies are generally subject to a more extensive review process, where observational study reviews may be more marginal. Nonetheless, duration was short in France and long in the UK. CENTER-TBI is registered as an observational study, in which ‘the investigator is not acting upon study participants, but instead observing natural relationships between factors and outcomes’ [19]. Two IRBs considered the study to be purely interventional. Interventional studies are studies ‘where the researcher intercedes as part of the study design’ [19]. An explanation for this opposing classification is that the IRBs did and did not consider the following procedures to be standard-of-care: (1) Different amounts of additional blood draws at presentation and follow-up. (2) Neuropsychological assessments and outcome questionnaires up to a 24-month follow-up. (3) Additional MRIs at sites participating in the MRI sub-study.

Extra work without clear benefits delays projects and should be avoided when possible. An additional IRB review after primary central IRB approval is usually double work and could result in an extra delay of weeks to more than a year, without always having clear benefits over the already obtained primary approval [17]. Cancelling potentially unnecessary (extensive) additional IRB review procedures could not only reduce turnover time, but also reduce costs. The exact costs of European IRB review procedures are unfortunately unknown, but the direct costs of an IRB review and approval in the US have been calculated to be $107.544 ($82.610 in IRB fees and $24.934 in labor) [20].

Delays in obtaining IRB approval not only adversely affect study initiation, but are also associated with several other risks. Long procedures with many feedback rounds will delay study start, frustrate researchers and might even endanger meeting subsidiary demands. Researchers might attempt to speed up the process by changing the protocol or submitting the protocol to IRBs that are considered to be less strict but able to process the submission the quickest. This does not necessarily serve primary research objectives and might even hamper quality and generalizability of study results.

Optimization of IRB review procedures is urgently needed as multinational collaborations in healthcare research are increasing and even promoted by multiple European research grant [4, 5, 21]. Harmonization and adequate implementation of regulatory and ethical standards between European countries could improve the present situation [7, 22]. The EU already aims to freely cooperate across borders by defining common standards and removing legal obstacles, but true harmonization of Member State laws in a research context has clearly not been established yet [21–24]. For example, the General Data Protection Regulation (GDPR) aimed to ensure a fair and transparent processing of personal data and aimed to improve patients’ control over their own data [25]. The implementation and use of the GDPR however showed the difficulty of harmonization in the protection of the EU citizens in this context. This was especially caused by the possibility for European countries to use their own national legislation in addition to the GDPR, which does not improve the desired harmonization.

Harmonization remains a highly complex process due to variation of national regulations that are based on national customs, culture, ethics, religion and other beliefs [6]. Harmonization of laws is designed to incorporate different legal systems under a basic framework. To overcome the highly complex process of harmonization in the area of research, it has been suggested to combine similarities between legislations and regulations of countries under a basic framework like a European research directive. A framework should acknowledge these local cultural or religious beliefs, as disregarding them is neither feasible nor desirable. While the desirable goal of harmonizing regulation will certainly benefit research in the future, both IRBs and researchers will have to put in efforts until that time. IRBs can accelerate the turnover by only requiring central IRB approval and researchers should respond quicker and more comprehensively to questions from IRBs, preventing the repetition of questions.

### Strengths and limitations

The CENTER-TBI study provides a unique opportunity to provide comprehensive insight in the procedural differences between European IRBs. The study benefits from its large size and because the data acquisition process increased the quality and completeness of documents. Despite the quality of the documents, results were still dependent on the recorded information. Therefore, we could not always identify causal factors for variation, which is something to look for in future initiatives. The data on IRB review procedures in an observational study conducted with mentally incapacitated patients in neurotrauma centers might not be generalizable for other research settings.

## Conclusions

This study shows variation between IRB procedures across Europe, which pose major challenges to large European research collaborations. Differences are likely caused by the lack of harmonization, integration and implementation of national legislations and regulations. To optimize efficiency for multinational European studies in context of obtaining IRB approval, the encountered differences and inefficiencies should be studied further and policymakers should evaluate the opportunities to optimize regulatory harmonization, while acknowledging the boundaries of national sovereignty and local cultural preferences.

## Supplementary information

**Supplementary files.**

## Data Availability

There are legal constraints that prohibit us from making all data publicly available. Data could be identifiable because the limited number of centres per country that were included in this study. Readers may contact Dr. Erwin J. O. Kompanje (erwinkompanje@me.com) for reasonable requests for the data.

## Abbreviations

EU: European Union
CENTER-TBI: Collaborative European NeuroTrauma Effectiveness Research in Traumatic Brain Injury
CER: Comparative Effectiveness Research
GDPR: General Data Protection Regulation
ICON: ICON plc
IRB: Institutional Review Board
M: Missing
PHD: Personal Health related Data
TBI: Traumatic Brain Injury
UK: United Kingdom
