# Supplementary material for: How do 66 European institutional review boards approve one protocol for an international prospective observational study on traumatic brain injury? Experiences from the CENTER-TBI study
Source: BMC Med Ethics. 2020 May 12;21:36. doi: 10.1186/s12910-020-00480-8 (PMC7216427; doi:10.1186/s12910-020-00480-8)
Supplement: Supplementary file 1 — Supplementary files. [file 12910_2020_480_MOESM1_ESM.docx]

**Supplementary Files**

| Overview of different types of IRB reactions | | |
| --- | --- | --- |
| Types of feedback | First review round  N =10 (%) | Second review round  N = 5 (%) |
| Procedure | 8 (80) | 2 (40) |
| Blood collection and biomarkers | 5 (50) | 1 (20) |
| MRI | 5 (50) | 0 (0) |
| Privacy and data security | 4 (40) | 0 (0) |
| Other or unknown | 1 (10) | 2 (40) |

| Translated examples from communication records | |
| --- | --- |
| Types of feedback | Examples of feedback |
| Procedure | IRB of country A:  *‘Indicate how much blood is collected at which time intervals, how many MRI examinations take place at which intervals, which tests and questionnaires must be completed how many times, the time required for study participation to be specified. The MRI examination should be described, if a contrast agent is used, this should be indicated, including the resulting risks. It is necessary to provide an overview of which patients' data are stored.”* |
|  | IRB of country B:  *‘The committee's reason for postponing final evaluation of the application was mainly directed at the planned inclusion of children, as well as some uncertainties related to the planned genetic studies and, if any, reuse of the material in other studies.’* |
| Blood collection and biomarkers | Researcher of country C  *‘In all patients, blood samples will be extracted through routine laboratory tests as well as standard clinical procedures. For the purposes of the study, all patients of all strata will obtain 19 ml extracted within 24 hours of the lesion, of which 9 ml will be for biomarker analysis and the other 10 ml will be for genetic analysis. The amount of blood that is extracted for the purposes of the research will not exceed 20 ml any day. The donation of sample does not imply any extra expense for the patient (or their legal representative).’* |
|  | IRB of country D  *‘Without the written consent a blood sample for the study could be taken in conjunction with other blood samples taken for normal care. (…) The requirement makes it impossible to conduct an acute phase of blood samples from critically ill patients, which is not in the interests of the investigators. If this is not the case, it is not possible to initiate the study in full (including a blood sample) only after written consent.’* |
| MRI | IRB of country E  *‘The collection of data on children who are taking MRI, can be considered as a non-intervention registration / quality assurance study, as long as the MRI is part of the normal treatment practice. Taking MRI additional to normal care cannot be approved as far as sedation is needed because the sedation itself can cause disadvantages and risks to the children. (…) Parents has to be informed that their child cannot enter the trial if the child need sedation during the MRI. The protocol has to outline the reasons for the inclusion of such participants in the MRI. There has to be available participant information for this group.’* |
|  | Researcher of country F  *‘About healthy research participant: Healthy volunteers are necessary to obtain reference values. Diffusion values are required for the advanced analysis of Diffusion Tensor Images. The total inclusion is limited to nine healthy volunteers. They are invited to undergo the MRI on a completely voluntary basis. The volunteers must be in the 18-65 age group. They will be informed in advance about the chance of discovering chance findings and can indicate whether they want to be informed of this or not.’* |
| Privacy and data security | IRB of country A  *‘Participants must be informed where the samples are stored and who (person or holder) is responsible for the storage and destruction of the samples. (…) It should be clarifying whether the samples are (in fact completely) anonymization, pseudonymization (encryption) participants can request that their samples be destroyed if they withdraw from the study. (…) The transfer of the samples to third parties, and in particular to partners from the industry, must be specified in more detail - at least the participants must be informed how they can always find out to whom their samples have been forwarded.’* |
|  | Researcher of country C  *‘The patient is informed of how their anonymous data will be stored safely, in accordance with good research practices. The patient is informed of how to proceed with the blood samples processed and transferred to these blood repositories. “The data obtained from the study will have all the personal identification information removed and transferred to a central repository where it will be analyzed to answer the research questions that are part of the CENTER-TBI. This anonymous data will be stored safely for a minimum of 6 years and possibly indefinitely in a secure data file in accordance with good research practices. The CENTER-TBI study will end in 2020. We plan to keep these blood and data repositories as a legality for future research. If this is not possible, we will seek to have the approval of responsibility to maintain the repositories to one of the research organizations in Europe to combine with other similar collections. If this is not possible, the residual samples and data will be destroyed.”’* |
